# TALKING ABOUT LONELINESS: QUALITATIVE INSIGHTS FROM OLDER ADULTS: IMPLICATIONS FOR RESEARCH, POLICY, AND SERVICES

**DOI:** 10.1093/geroni/igac059.613

**Published:** 2022-12-20

**Authors:** Roger O'Sullivan, Gerry Leavey

## Abstract

The very personal and complex nature of loneliness is too rarely articulated in research papers. Each presenter in this interdisciplinary and international symposium presents insights into loneliness and /or social isolation that can help bridge this gap. Victor (Social Gerontology) using open ended responses from the 2018 BBC Loneliness Experiment, presents how 1480 older people describe loneliness and highlights the need to give more attention to existential loneliness. O’Sullivan (Public Health) presents the results of 18 life story interviews with older adults attending a mental health service. The analysis identified three different typologies of loneliness with specific recommendations for training and services. Phone-based support programs are increasingly being used as a solution for those experiencing loneliness. However, less is known about what aspects are most helpful. Perissinotto (Geriatrics and Palliative care) presents results from 38 qualitative interviews with a focus on barriers and facilitators to implementing a phone-based support intervention, particularly for older adults experiencing loneliness. Cudjoe (Medicine) presents qualitative data from older adults (English, Spanish and Mandarin speaking), living in non-profit affordable housing in 22 different states. Drawing on experiences of their social connections during the COVID-19 pandemic, the paper gives voice to the implications of the loss of common facilities, and opportunities to socialize with other residents, and the increased role technology plays in staying connected. Our discussant, Prof Leavey, a leader in the field of mental health, will reflect on the major themes emerging from these multidisciplinary perspectives, especially what they mean for public health and services.

